# Abnormal Calcium Handling and Exaggerated Cardiac Dysfunction in Mice with Defective Vitamin D Signaling

**DOI:** 10.1371/journal.pone.0108382

**Published:** 2014-09-30

**Authors:** Sangita Choudhury, Soochan Bae, Qingen Ke, Ji Yoo Lee, Sylvia S. Singh, René St-Arnaud, Federica del Monte, Peter M. Kang

**Affiliations:** 1 Cardiovascular Institute, Beth Israel Deaconess Medical Center and Harvard Medical School, Boston, Massachusetts, United States of America; 2 Shriners Hospital and Departments of Surgery and Human Genetics, McGill University, Montreal, Canada; Texas A & M University Health Science Center, United States of America

## Abstract

**Aim:**

Altered vitamin D signaling is associated with cardiac dysfunction, but the pathogenic mechanism is not clearly understood. We examine the mechanism and the role of vitamin D signaling in the development of cardiac dysfunction.

**Methods and Results:**

We analyzed 1α-hydroxylase (1α-OHase) knockout (1α-OHase^−/−^) mice, which lack 1α-OH enzymes that convert the inactive form to hormonally active form of vitamin D. 1α-OHase^−/−^ mice showed modest cardiac hypertrophy at baseline. Induction of pressure overload by transverse aortic constriction (TAC) demonstrated exaggerated cardiac dysfunction in 1α-OHase^−/−^ mice compared to their WT littermates with a significant increase in fibrosis and expression of inflammatory cytokines. Analysis of calcium (Ca^2+^) transient demonstrated profound Ca^2+^ handling abnormalities in 1α-OHase^−/−^ mouse cardiomyocytes (CMs), and treatment with paricalcitol (PC), an activated vitamin D_3_ analog, significantly attenuated defective Ca^2+^ handling in 1α-OHase^−/−^ CMs. We further delineated the effect of vitamin D deficiency condition to TAC by first correcting the vitamin D deficiency in 1α-OHase^−/−^ mice, followed then by either a daily maintenance dose of vitamin D or vehicle (to achieve vitamin D deficiency) at the time of sham or TAC. In mice treated with vitamin D, there was a significant attenuation of TAC-induced cardiac hypertrophy, interstitial fibrosis, inflammatory markers, Ca^2+^ handling abnormalities and cardiac function compared to the vehicle treated animals.

**Conclusions:**

Our results provide insight into the mechanism of cardiac dysfunction, which is associated with severely defective Ca^2+^ handling and defective vitamin D signaling in 1α-OHase^−/−^ mice.

## Introduction

Cardiovascular disease (CVD) is the most common cause of mortality and morbidity in the United States and other developed nations. Clinical and epidemiological studies suggest an association between vitamin D deficiency and various cardiovascular disorders [1], [2], and vitamin D therapy has been shown to improve cardiovascular function [3]. Particularly, in patients with chronic renal failure (CRF), vitamin D deficiency is uniformly present because the critical conversion of nutritional vitamin D_3_ (25(OH)D_3_) to the hormonally active form of vitamin D_3_ (1,25(OH)_2_D_3_) occurs primarily in the kidney by 1α-hydroxylase (1α-OHase)[4]. CRF has been shown to be an independent risk factor for CVD, with 10–20 times greater incidence of cardiovascular disease in CRF patients [5]. Clinical studies have also demonstrated that there is an association between improved survival and decreased cardiovascular mortality in hemodialysis patients treated with an activated vitamin D analog, paricalcitol (PC) [6], [7]. In fact, similar to CRF patients, 1α-OHase knockout (1α-OHase^−/−^) mice show baseline hypertension, cardiac hypertrophy, and impaired cardiac function [8]. Despite these associations between vitamin D deficiency and cardiac dysfunction, the pathogenic mechanism of cardiac dysfunction associated with vitamin D deficiency is not fully elucidated.

Cardiomyocyte (CM) contractility is regulated by Ca^2+^ handling contractile proteins via regulation of intracellular levels of Ca^2+^ [9]. Thus, abnormalities in Ca^2+^ homeostasis, as seen in vitamin D deficiency, may lead to a chronic defect in E–C coupling, which may in turn lead to cardiac dysfunction. Delays in Ca^2+^ transients, for example, have been observed in myocardial tissue obtained from failing hearts [10]. Although it might not be surprising to find abnormal Ca^2+^ handling in a vitamin D deficiency state, the exact mechanism of vitamin D deficiency affecting the Ca^2+^ handling in CMs, however, is poorly understood. In this study, we examined whether abnormal Ca^2+^ homeostasis and Ca^2+^ handling may mediate structural and functional cardiac abnormalities and examined the relationship between vitamin D deficiency and the development of cardiac hypertrophy in 1α-OHase^−/−^ mice. We hypothesize that restoration of vitamin D signaling improves cardiac functions by restoring Ca^2+^ homeostasis.

## Methods

### Animal care

Experiments were conducted using 8–10-week old male 1α-OH^−/−^ mice and their littermates in a C57BL/6 background. 1α-OHase^+/−^ and 1α-OHase^−/−^ mice were produced from 1α-OHase^+/−^ [11]. All mice, breeders and offspring were housed at the Animal Research Facility at Beth Israel Deaconess Medical Center (BIDMC) under pathogen-free conditions with a reverse daily 12∶12 h light: dark cycle. Euthanasia was performed by CO_2_ via a gas cylinder. All experimental procedures were approved by the Institutional Animal Care and Use Committee of BIDMC.

### Animal surgery and hemodynamic measurement

TAC was performed in 8–10 week old male C57BL/6 mice and in 1α-OHase^−/−^ [12], [13] mice. Cardiac function was analyzed using echocardiography (for baseline cardiac function) and left ventricular (LV) pressure-volume loop measurement (after TAC) as described previously [14], [15]. Detailed method is provided in Text S1.

### Morphometric analysis of isolated CMs

CMs were enzymatically dissociated from 12 weeks old male mouse hearts according to previously described protocol [16], [17]. Detailed method is provided in Text S1.

### Serum Ca^2+^ and vitamin D metabolite analysis

Serum 1, 25(OH)_2_ D_3_ (n = 8–10 replicates per group) was analyzed via enzyme immunoassays using commercial kits (Immunodiagnostic Systems). Serum Ca^2+^ (n = 8–10) was analyzed via a quantitative Calcium Colorimetric Detection Kit (Bio Vision).

### Serum PTH analysis

Serum PTH (n = 8–10 replicates per group) was analyzed via enzyme immunoassays using commercial kits (Immunooptics Inc.)

### Ca^2+^ transients and cell shortening measurements of isolated CMs

Measurement of Ca^2+^ transient and cell shortening in isolated CMs [18]. Briefly, CMs were isolated and contraction parameters and Ca^2+^ transient [Ca^2+^] i were measured in response to electrical stimulation. Caffeine induced [Ca^2+^] i release was measured after a 10-second pause following steady-state stimulation at increasing rates. Detailed methods are described in Text S1.

### Histology and Western Blots

Hearts were fixed in 10% formalin and paraffin-embedded. Sections were stained with hematoxylin and eosin and Masson Trichrome (MT) at the Histology Core facility at BIDMC [14], [15]. Quantification of fibrosis was performed as described previously [16]. To compare the levels of the major proteins involved in Ca^2+^ handling, Western blot analysis was performed on total protein from WT and 1α-OHase^−/−^ mice as described previously [14], [15].

### Statistical Analysis

Data are reported as mean SEM. Statistical significance was determined by one-way or two-way ANOVA and Student-Newman-Keuls post hoc test. Values of *p*<0.05 were considered significant.

## Results

### Exaggerated hypertrophic responses after transverse aortic constriction (TAC) in 1α-OHase^−/−^ mice

**Figure 1:**
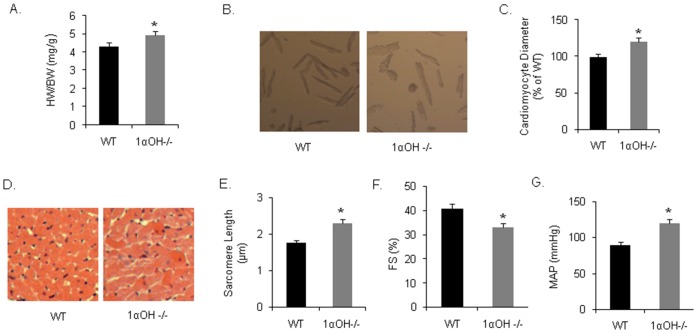
Physiological, echocardiographic, and hemodynamic parameters in 1αOHase−/− mice at baseline: (A) Baseline HW/BW of 1α-OHase −/− mice. *, *p*<0.05 compared to WT. N = 8–10. (B and C) Isolated adult CMs from 1α-OHase^−/−^ mice showing increased surface area compared to WT CMs. *, *p*<0.05 compared to WT. N = 8–10. (D) Cross sectional area of mice heart showing CM surface area. (E) Resting sarcomere length of 1α-OHase^−/−^ and WT CMs. *, *p*<0.05 compared to WT. N = 8–10. (F) Baseline fractional shortening (FS) of WT and KO mice. *, *p*<0.05 compared to WT. N = 8–10. (G) MAP of WT and 1α-OHase−/− mice at baseline. *, *p*<0.05 compared to WT. N = 8–10.

At baseline, 1α-OHase^−/−^ mice demonstrated a modest 5% increase in heart weight compared to their WT littermates at 12 weeks of age (**Fig. 1A**). Corresponding isolated adult CMs also demonstrated modest increase in surface areas of 1α-OHase^−/−^ mice compared to the age and gender matched WT mice (**Fig. 1B and 1C**). Cross sectional area of the CMs further confirmed these findings (**Fig. 1D and 1E**). We also observed that baseline systolic cardiac function was modestly reduced in 1α-OHase^−/−^ mice compared with the WT mice (**Fig. 1F**). In addition, the mean arterial pressure (MAP) of 1α-OHase^−/−^ was significantly higher than that of WT littermate mice (**Fig. 1G**). These findings suggested that there is baseline mild cardiac hypertrophy and cardiac dysfunction in 1α-OHase^−/−^ mice compared to the WT mice.

**Figure 2:**
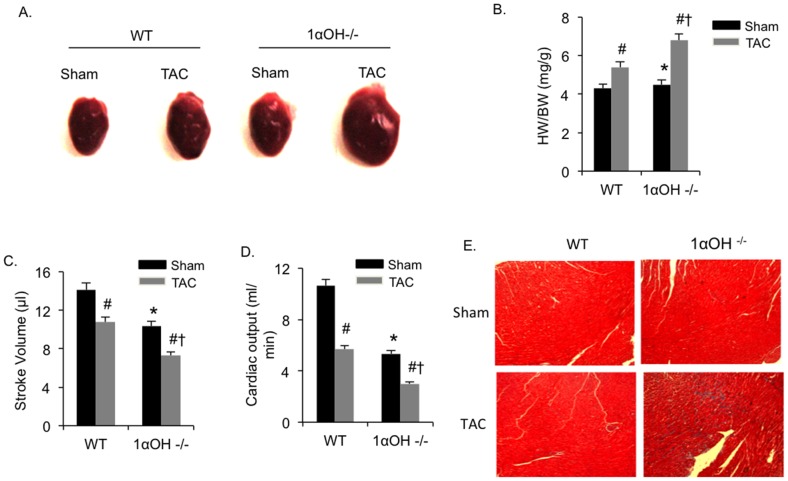
In response to pressure overload mutant mice showing exaggerated hypertrophy. (A) Representative hearts after 4 weeks of sham or TAC in WT and 1α-OHase^−/−^ mice. (B) HW/BW after 4 weeks of sham or TAC in WT and 1α-OHase^−/−^ mice. *, *p*<0.05 compared to WT. #, *p*<0.05 compared to sham. †, *p*<0.05 compared to WT TAC. N = 8–10. (C and D) Stroke volume (C) and cardiac output (D) in WT and 1α-OHase^−/−^ mice after 4 weeks of sham or TAC. *, *p*<0.05 compared to WT. #, *p*<0.05 compared to sham. †, *p*<0.05 compared to WT TAC. N = 8–10. (E) Representative Masson-Trichrome heart staining in 1α-OHase^−/−^ mice after 4 weeks of sham or TAC.

To elucidate the effect of defective vitamin D signaling under pathological conditions, we imposed pressure overload on the heart using TAC. Morphometric analysis demonstrated significant increases in heart weight/body weight (HW/BW) ratio in both 1α-OHase^−/−^ and WT mice compared with the corresponding sham-operated groups after 4 weeks of TAC (**Fig. 2A and 2B**). However, there were exaggerated hypertrophic responses to TAC in 1α-OHase^−/−^ mice heart compared to the WT mice. Cardiac functional analysis using pressure-volume (PV) loop measurement revealed that 1α-OHase^−/−^ mice demonstrated significantly greater cardiac dysfunction after TAC, as observed by reduction in stroke volume and cardiac output, compared to their WT littermates (**Fig. 2C and 2D**).

We further analyzed these hearts for the presence of interstitial fibrosis using Masson-Trichrome staining We found that both WT and 1α-OHase^−/−^ mice showed presence of fibrosis after TAC, (**Fig. 2E**) which was significantly increased in 1α-OHase^−/−^ mice compared with those in WT mice (WT  = 11.9±1.5% vs. 1α-OHase^−/−^  = 21.5±1.2%; *p*<0.05). These findings demonstrated that there were exaggerated pathological responses to TAC in 1α-OHase^−/−^ mice that are associated with greater decreased cardiac function and more aggressive development of cardiac fibrosis.

### Increased fetal gene activation and inflammatory responses after TAC in 1α-OHase^−/−^ mice

**Figure 3:**
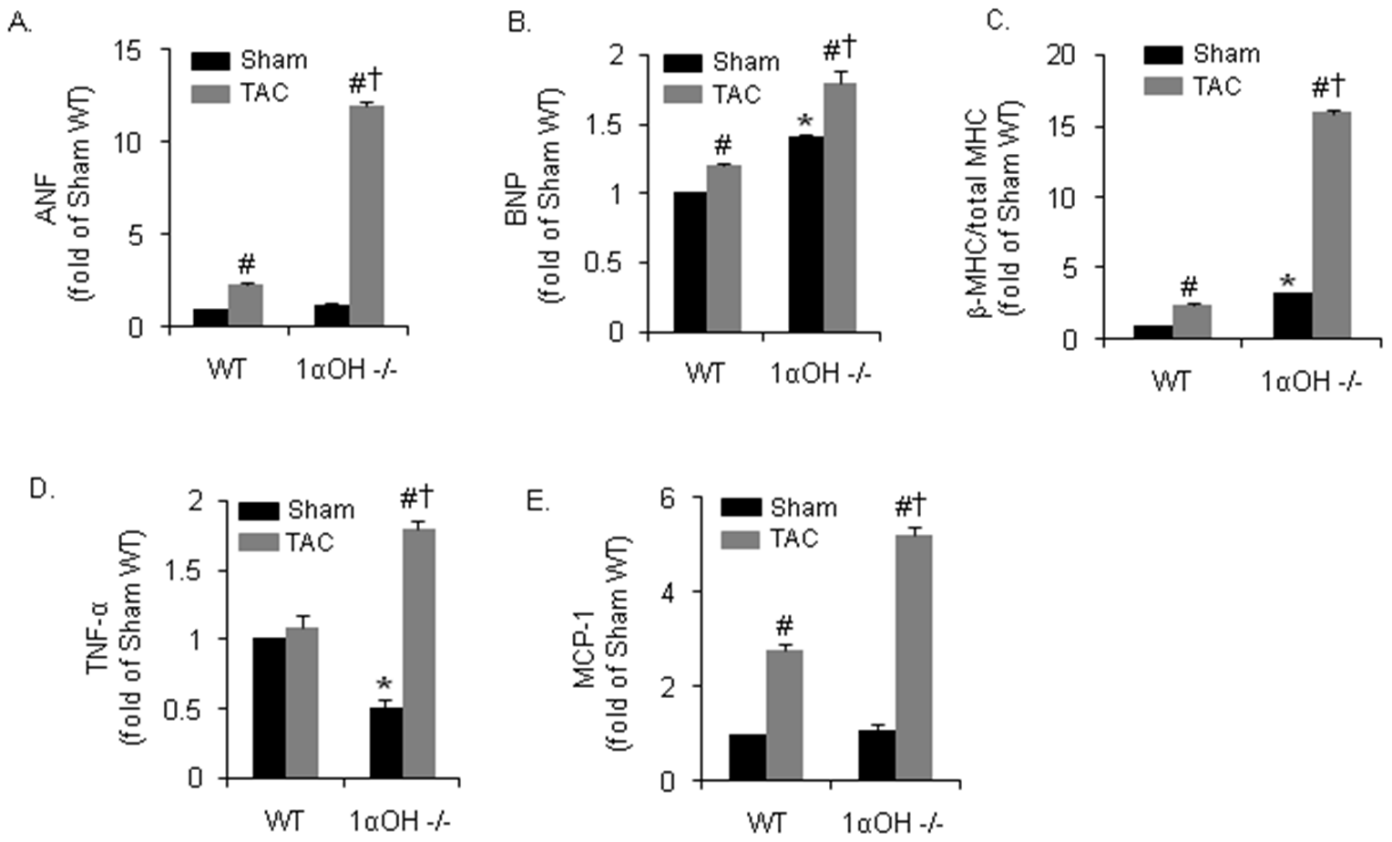
Biochemical findings after 4 weeks of Pressure overload in 1αOHase^−/−^ mice. (A–C) Real-time PCR mRNA expression of ANF (A), BNP (B) and of ß-MHC to total MHC ratio (C) in WT and 1α-OHase^−/−^ mice after 4 weeks of sham or TAC. *, *p*<0.05 compared to WT. #, *p*<0.05 compared to sham. †, *p*<0.05 compared to WT TAC. N = 8–10. (D and E) Real-time PCR mRNA expression of inflammatory cytokines TNF-α (D) and MCP-1 (E) in WT and 1α-OHase -/- mice after 4 weeks of sham or TAC. *, *p*<0.05 compared to WT. #, *p*<0.05 compared to sham. †, *p*<0.05 compared to WT TAC. N = 8–10.

We determined the biochemical markers for cardiac hypertrophy and heart failure by measuring the level of “fetal” genes, such as atrial natriuretic factor (ANF) and brain natriuretic peptide (BNP). Ventricular ANF was significantly expressed in all TAC groups. However, there was a 8-fold greater increase in ANF mRNA levels after TAC in 1α-OHase^−/−^ mice compared to the WT littermates (**Fig. 3A**). Similarly, there was also a greater increase in BNP expression in 1α-OHase^−/−^ mice compared to the WT mice hearts after TAC (**Fig. 3B**). Another marker for cardiac hypertrophy, the ratio of ß-MHC to total MHC mRNA expression, was also increased after TAC in both groups. Yet, there were again dramatic and exaggerated increases observed in 1α-OHase^−/−^ mice compared to the WT mice after TAC (**Fig. 3C**).

It's known that vitamin D, a steroid hormone, affects immune regulation by preventing excessive expression of inflammatory cytokines and increasing the ‘oxidative burst’ potential of macrophages [19]. Interestingly, we observed a reduction in tumor necrosis factor-α (TNF-α) expression at baseline in 1α-OHase^−/−^ mice compared to WT mice. Although no significant differences were observed in TNF-α expression after TAC in WT mice, 1α-OHase^−/−^ mice hearts showed enhanced activation of TNF-α after TAC (**Fig. 3D**). Another inflammatory marker, monocyte chemotactic protein-1 (MCP-1), was activated by TAC in both mice, but with significantly greater activation was observed in 1α-OHase^−/−^ mice after TAC (**Fig. 3E**). These findings demonstrated that there is an exaggerated biochemical evidence of cardiac dysfunction and uncontrolled immune response associated with TAC in 1α-OHase^−/−^ mice.

### Defective Ca^2+^ handling and modulation of Ca^2+^ regulatory protein expressions in 1α-OHase^−/−^ mice CMs

**Figure 4:**
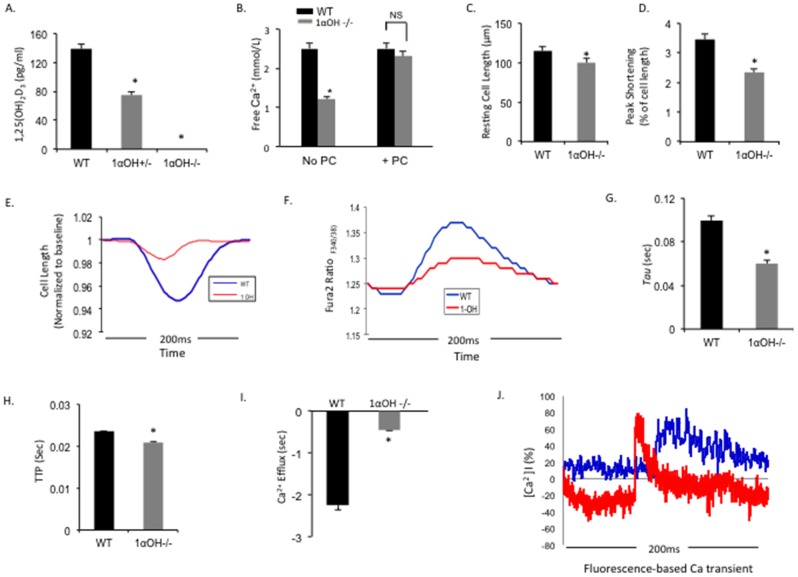
Contractile function and Ca^2+^ transient parameters in isolated CMs from 1αOHase −/− mice. (A and B) Baseline 1, 25(OH)_2_D_3_ (A) and free Ca^2+^ (B) level in WT and 1αOHase −/− mice serums. *, *p*<0.05 compared to WT, N = 6–8 mice. (C) Resting cell length (D) and peak shortening (% of cell length) from isolated CMs obtained from WT and 1αOHase −/− mice. *, *p*<0.05 compared to WT. Ten twitches per CMs were collected for each mouse heart. N = 6–8 mice/group. (E) Continuous measurement of cell contractility from isolated CMs obtained from WT (blue line) and 1αOHase^−/−^ (red line) mice. *, *p*<0.05 compared to WT. Ten twitches per CMs were collected for each mouse heart. N = 6–8 mice/group. (F) Representative Fura 2 ratio (F340/380) from isolated CMs obtained from WT (blue line) and 1αOHase -/- (red line) mice. Data shows that the twitch peak amplitudes are significantly different between WT and 1αOHase -/- CMs, which indicate severely defective Ca^2+^ handling in 1αOHase^−/−^ CMs. (G–I) The rate of Ca^2+^ transient decay (*Tau*) (G), time to peak contraction (TTP) (H), and return velocity to baseline or Ca^2+^ efflux in WT and 1αOHase^−/−^ CMs. *, *p*<0.05 compared to WT. N = 10 CMs/mouse heart. N = 6–8 mice/group. (J) Representative traces of Ca^2+^ transients evoked by 10 mM caffeine recorded in WT (blue line) and 1αOHase^−/−^ (red line) mice CMs.

We found that the circulating 1, 25(OH) vitamin D_3_ level was undetectable in 1α-OHase^−/−^ mice and significantly reduced in heterozygous 1α-OHase^+/−^ littermates (**Fig. 4A**), which coincide with previous findings [20], [21]. Without vitamin D supplementation, 1α-OHase^−/−^ mice at 12 weeks showed severe hypocalcemia with plasma Ca^2+^ concentrations as low as 1.2 mmol/L in contrast to the WT littermates, which exhibited normal plasma Ca^2+^ concentrations (WT = 2.5 mmol/L vs. 1α-OHase^−/−^  = 1.2 mmol/L; P<0.05). Supplementation of vitamin D using injection of PC (200 ng/kg), an activated vitamin D_3_ analog, starting at 4 weeks after birth normalized the plasma Ca^2+^ concentration in 1α-OHase mice by 12 weeks (**Fig. 4B**).

To examine the effect of the vitamin D deficiency in CMs, contraction parameters of individual isolated CM from 1α-OHase^−/−^ mice and their WT littermates were studied by measuring sarcomere shortening (contraction) and relengthening (relaxation) in response to electrical stimulation (**see Text S1 for detail**). Baseline 1α-OHase^−/−^ CM sarcomeres were significantly larger compared to WT (**Fig. 4C** **, Table S1**). Additionally, 1α-OHase^−/−^ CMs showed a significant decrease in peak shortening, as well as the rate of cell shortening and rate of cell relengthening compared to WT CMs (**Fig. 4D and 4E**). Peak shortening is calculated from the percent shortening of length data (**Fig. 4D**). The highest and lowest value reached by the transient.

We then investigated Ca^2+^ handling properties of individual CM from 1α-OHase^−/−^ mice (**see Text S1 for detail**). Analysis of Ca^2+^ transients indicated decreased peak systolic Ca^2+^, measured as Fura-2 ratios in CM from 1α-OHase^−/−^ compared to WT group CM (**Fig. 4F**). The rate of Ca^2+^ transient decay (*tau,* exponential decay of time constant, the speed of relaxation/calcium uptake) was also significantly reduced in 1α-OHase^−/−^ CMs (**Fig. 4G**) which was calculated by the ionoptix program from the transient, with a reduced time to peak (TTP) and a decrease in relaxation rate (Ca^2+^ efflux) (**Fig. 4H and 4I**). Caffeine causes Ca^2+^ release from the sarcoplasmic reticulum (SR) of mammalian muscle. Thus, to evaluate the SR Ca^2+^ load, we tested caffeine-induced Ca^2+^ release amount from SR in WT and 1α-OHase^−/−^ CMs. We found that CMs from 1α-OHase^−/−^ mice presented a decreased caffeine-induced Ca^2+^ transient compared to WT CMs (**Fig. 4J**). These findings demonstrated profound Ca^2+^ handling abnormalities in 1α-OHase^−/−^ mice CMs, which may contribute to the development of fulminant heart failure after TAC.

**Figure 5:**
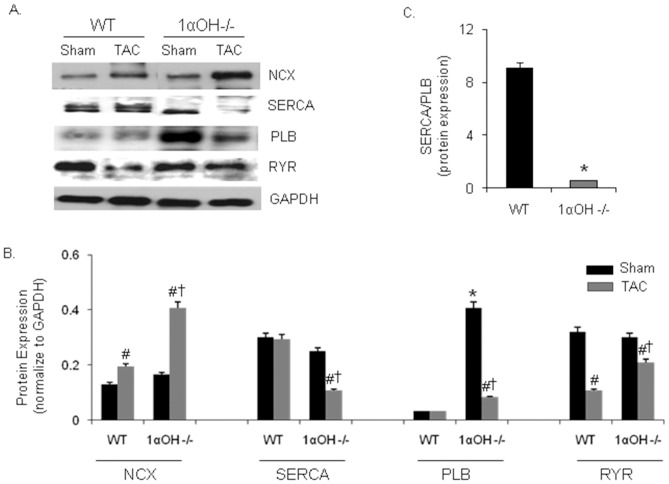
Expressions of Ca^2+^ regulatory protein in mice with altered metabolism of Vitamin D. (A) Representative western blots of various Ca^2+^ handling proteins. (B) Quantitative analysis of various Ca^2+^ handling proteins. NCX  = Na^+^/Ca^2+^ exchanger, SERCA2a  =  sarcoplasmic reticulum Ca^2+^ ATPase, PLB  =  phospholamban, and RYR  =  ryanodine receptor. GAPDH  =  glyceraldehyde 3-phosphate dehydrogenase. GAPDH was used as internal loading control. *, *p*<0.05 compared to WT. #, *p*<0.05 compared to sham. †, *p*<0.05 compared to WT TAC. N = 8–10. (C) Quantitative analysis of SERCA/PLB protein expression ratio. *, *p*<0.05 compared to WT. #, p<0.05 compared to sham, N = 8–10.

The expression level and the activity of sarcoplasmic reticulum Ca^2+^ ATPase (SERCA) is significantly decreased in pressure overload-induced hypertrophy and during heart failure in animals and human, which correlate with decreased myocardial dysfunction [22], [23]. To further elucidate the mechanism underlying the defective Ca^2+^ handling, we examined the SERCA2a protein expression level in these mice. We found that 1α-OHase^−/−^ mice showed significant decreased SERCA2a expression after TAC compared to WT mice (**Fig. 5A and 5B**). Phospholamban (PLB) interacts with SERCA and inhibits its Ca^2+^ transport rate, and the relative abundance of SERCA and PLB maintains Ca^2+^ homeostasis and CM contractility. Therefore, SERCA/PLB ratio reflects Ca^2+^ transport capacity of the SR [24]. At baseline, 1α-OHase^−/−^ mouse hearts showed significant increase in PLB, which decreased after TAC (**Fig. 5A and 5B**). SERCA/PLB ratio was also significantly decreased in 1α-OHase^−/−^ mice compared to WT mice (**Fig. 5C**). This may explain the difference in SR Ca^2+^ load and contractile function between WT and 1α-OHase^−/−^ mice CMs, and the depressed levels of SERCA2a may contribute to the severity of heart failure after TAC in 1α-OHase^−/−^ mice.

The Na^+^- Ca^2+^-exchanger (NCX) is the dominant myocardial Ca^2+^ efflux mechanism. Ca^2+^ removal from the cytosol occurs by activity of the SR Ca^2+^ pump and by exchange of Ca^2+^ for sodium by the sarcolemmal NCX [25]. The decrease in PLB might lead to a higher activation of the remaining SERCA pumps and increase the activity of NCX [26]. At baseline, there were no significant difference in expression of NCX between the WT and 1α-OHase^−/−^ mice (**Fig. 5A and 5B**). However, we found significant up-regulation of NCX expression in banded WT and 1α-OHase^−/−^ mice with significant 4-folds increase seen in 1α-OHase^−/−^ mice. A relative increase in NCX is expected when SR function is impaired [27]. The function of ryanodine receptor 2 (RyR2) (the cardiac isoform) is to allow Ca^2+^-induced Ca^2+^ release that brings about contraction, while myocyte relaxation results in RyR2 closure accompanied by the Ca^2+^ re-uptake into SR through the SERCA [28]. After TAC, there was a moderate decrease in RyR2 expression in both WT and 1α-OHase^−/−^ mice (**Fig. 5A and 5B**).

### Treatment with PC attenuates cardiac dysfunction after TAC in 1α-OHase -/- mice

**Figure 6:**
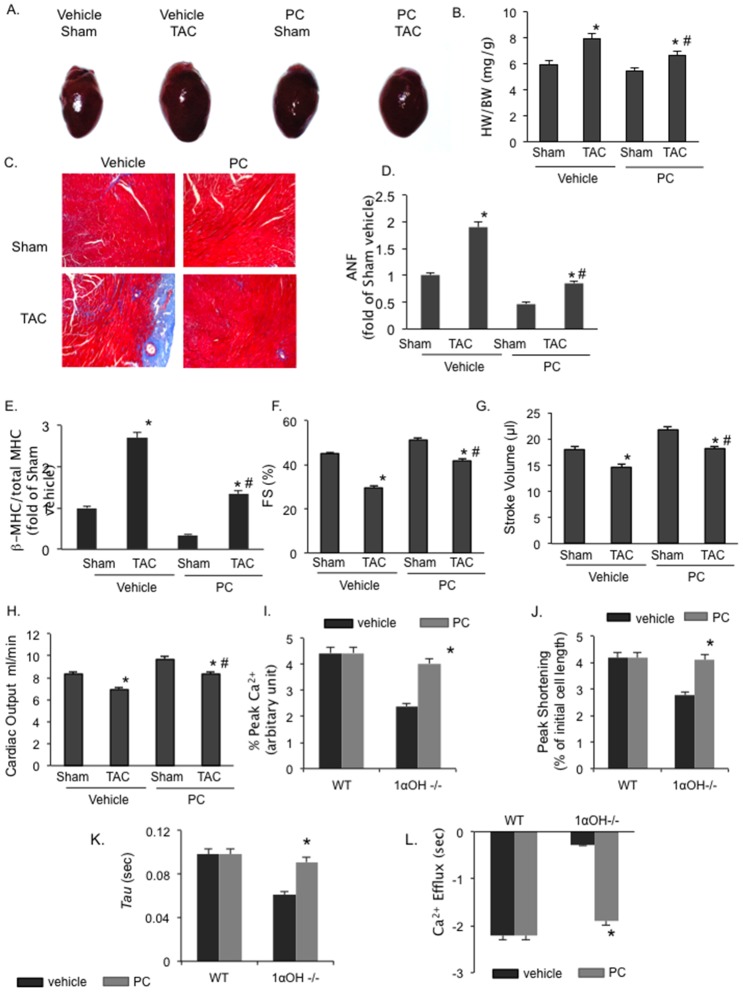
Effect of paricalcitol on cardiac dysfunction parameters. (A) Representative hearts in 1α-OHase^−/−^ mice with or without PC treatment after 4 weeks of sham or TAC. (B) HW/BW ratio in 1α-OHase^−/−^ mice with or without PC treatment after 4 weeks of sham or TAC. *, *p*<0.05 compared to Sham. #, *p*<0.05 compared to vehicle TAC. N = 8–10. (C) Representative Masson-Trichrome heart staining in 1α-OHase^−/−^ mice with or without PC treatment after 4 weeks of sham or TAC. (D and E) Real-time PCR mRNA expression of ANF (D) and of ß-MHC to total MHC ratio (E) after 4 weeks of sham or TAC in 1α-OHase^−/−^ mice with or without PC treatment. *, *p*<0.05 compared to Sham. #, *p*<0.05 compared to vehicle TAC. N = 8–10. (F–H) Fractional shortening (F), stroke volume (G) and cardiac output (H) in 1α-OHase -/- mice with or without PC treatment after 4 weeks of sham or TAC. *, *p*<0.05 compared to Sham. #, *p*<0.05 compared to vehicle TAC. N = 8–10. Effect of paricalcitol on defective calcium handling in 1αOHase −/− mice. (I–L). The % peak Ca^2+^ (I), % peak shortening (J), the rate of Ca^2+^ transient decay (*Tau*) (K), and the return velocity to baseline or Ca^2+^ efflux (L) in 1α-OHase^−/−^ mice with or without PC treatment. *, *p*<0.05 compared to vehicle treated mice group, N = 20 CMs/mouse from 6–8 mice/group.

The responses of 1α-OHase^−/−^ mice to TAC in our previous experiments may be compounded by their baseline cardiac hypertrophy. Thus, we further determined the specific role of defective vitamin D to pathological conditions by first using “rescue” vitamin D protocol as described previously in 1α-OHase^−/−^ mice, where vitamin D deficiency and its non-cardiac phenotype were both corrected [29]. From 28–30 days of life, 1-α-OHase^−/−^ mice were treated with an initial “rescue” dose of 500 ng/kg of PC, followed by a daily “maintenance” dose of 200 ng/kg from 5 to 8 weeks of age. At 8 weeks, the animals were divided into the following groups: 1) Vehicle + Sham, 2) Vehicle + TAC, 3) PC + Sham, and 4) PC+TAC. For the vehicle conditions, maintenance doses of PC were changed to vehicle injections, while the PC dose was maintained for PC groups. TAC resulted in a significant increase in HW/BW and LW/BW ratios in mice receiving vehicle for 4 weeks compared to the sham operated mice (**Fig. 6A and 6B**). In mice treated with PC, there was a significant 18% attenuation of TAC-induced HW/BW ratio increase compared to the vehicle treated animals. Histological examination demonstrated that interstitial fibrosis induced by TAC in vehicle-administered 1α-OHase^−/−^ mice were significantly inhibited in PC treated group after TAC (**Fig. 6C**). TAC-induced increases in ANF and β-MHC gene expression, which were both attenuated by PC treatment (**Fig. 6D and 6E**).

We found that decreased fractional shortening after TAC in vehicle treated mice was significantly improved with vitamin D replacement in 1α-OHase^−/−^ mice (**Fig. 6G**). In addition, we observed reduced stroke volume and cardiac output in vehicle treated group after TAC. These parameters also improved after vitamin D replacement (**Fig. 6G and 6H**). Thus, vitamin D replacement resulted in significant attenuation of cardiac dysfunction after TAC in 1α-OHase^−/−^ mice.

### Treatment with PC attenuates defective Ca^2+^ handling associated with defective vitamin D signaling

**Figure 7:**
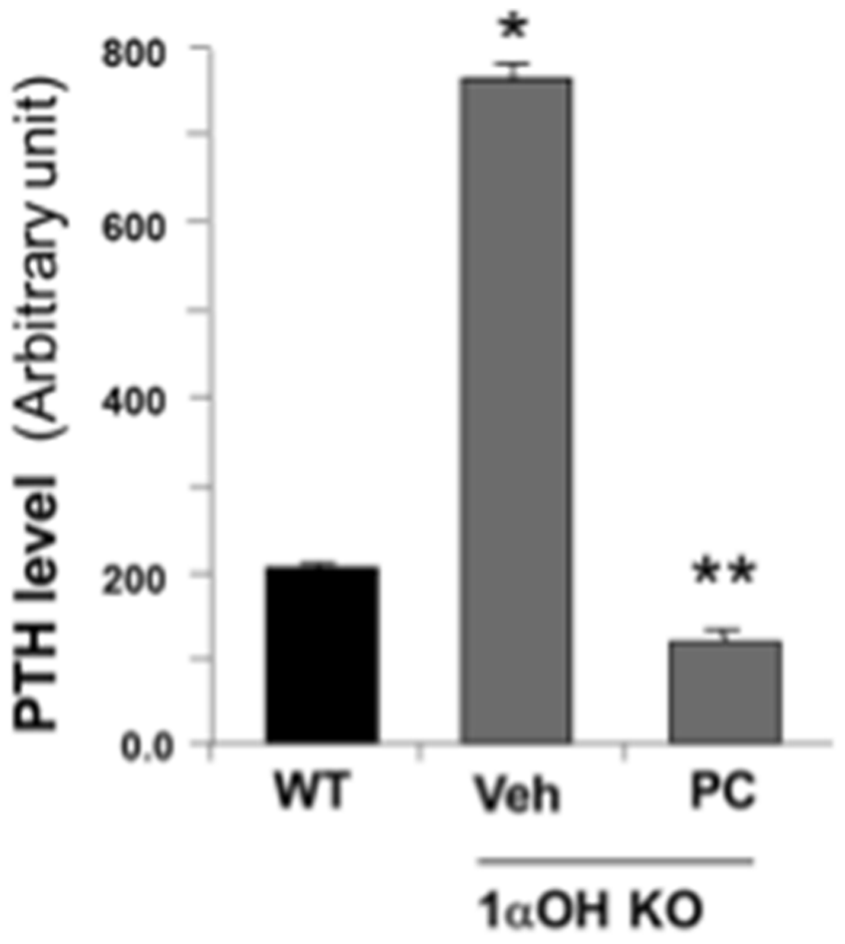
Paricalcitol restore the parathyroid hormone in 1α-OHase^−/−^ mice this model. PTH level in 1α-OHase^−/−^ mice with or without PC treatment. *, *p*<0.05 compared to WT. **, *p*<0.05 compared to vehicle. N = 8–10.

To further assess the effect of vitamin D replacement on CMs contractility in 1α-OHase^−/−^ mice contraction parameters, we performed Ca^2+^ handling analysis of isolated CMs in1α-OHase^−/−^ mice with or without PC treatment. We found that vitamin D replacement attenuated hypocontractility and increased peak shortening in PC treated 1α-OHase^−/−^ mice (**Fig. 6I and 6J**). Rate of relaxation was significantly increased with PC treatment and the rate of Ca^2+^ transient decay (*tau*) was significantly improved (**Fig. 6K and 6L**). Treatment with PC also normalized the PTH level in 1α-OHase^−/−^ mice (**Fig. 7**). These findings suggest that the pathological response in 1α-OHase^−/−^ mice is mainly due to defective vitamin D signaling and associated Ca^2+^ handling, and correcting these abnormalities could rescue these pathological responses.

## Discussion

In this study, we demonstrated that vitamin D deficiency is associated with cardiac hypertrophy at baseline results in an exaggerated progression to heart failure after pressure overload in 1α-OHase^−/−^ mice. We also found that there is defective Ca^2+^ handling in 1α-OHase^−/−^ mice, with structural and functional cardiac abnormalities in these mice. These cardiac Ca^2+^ handling abnormalities were completely corrected with vitamin D replacement in 1α-OHase^−/−^ mice. Thus, our studies strongly support the notion that vitamin D deficiency is an under-recognized, non-classic risk factor for developing heart failure that is readily correctable.

Vitamin D is known for its primary role in Ca^2+^ and bone homeostasis [30]. Biological activities of vitamin D are mediated by a hormonally active 1, 25-dihydroxyvitamin D_3_ that is converted from 25-hydroxyvitamin D_3_ by 1α-OHase. It binds to a specific high-affinity vitamin D receptor (VDR), a member of the superfamily of nuclear receptors for steroid hormones [4]. Vitamin D plays an important physiological role in controlling cardiac functions and vitamin D-dependent signaling systems are present in cardiac myocytes and fibroblasts [31]. Vitamin D deficiency has been associated with abnormal cardiac relaxation, proliferation, and increased cardiac renin gene expression [32], [33]. Previously, we showed that vitamin D therapy prevents the progression to cardiac hypertrophy [34], and attenuates the development of heart failure in salt sensitive rat model [35]. In addition, 1α-OHase -/- mice developed hypertension, cardiac hypertrophy, and impaired cardiac systolic function, possibly due to the activation of the RAS [8]. Clinically, vitamin D deficiency has been associated with the increased prevalence of myocardial dysfunctions and heart failure [36], [37]. Since the critical conversion of the storage form to the active form of vitamin D by 1α-OHase occurs in the kidney, patients with CKD are typically vitamin D deficient. In fact, the prevalence of cardiac hypertrophy and cardiac dysfunction is over 80% in these patients [38], [39], and vitamin D therapy has been shown to improve survival and decrease cardiovascular mortality [6], [7]. These findings suggest that the role of vitamin D signaling may be significant in the heart, and vitamin D deficiency may offer novel therapeutic target in the treatment of heart failure.

CM contraction is regulated by the interplay between Ca^2+^, contractile proteins, and the intracellular handling of Ca^2+^, whereas abnormal Ca^2+^ homeostasis is primarily responsible for depression of CM contractility [22], [23]. In several animal and human models of cardiac hypertrophy and heart failure, the whole-cell [Ca^2+^]_i_ transient is altered [40], [41]. Decreases in SERCA pump expression and activity have been observed in a variety of animal and human models of heart failure and a degree of decrease in SERCA level and its activity, closely correlate with a decreased myocardial function [27]. Thus, these data suggest that alterations in Ca^2+^ handling proteins are important contributors to cardiac dysfunction and heart failure. We found profound Ca^2+^ transient abnormalities, contractile abnormalities and altered expression of various Ca^2+^ handling regulatory proteins in vitamin D deficient CMs, which became exaggerated after TAC. Abnormalities in Ca^2+^ homeostasis, as seen in vitamin D deficiency, may lead to a chronic defect in E–C coupling, which may in turn lead to cardiac dysfunction. Particularly, decreased SERCA and SERCA/PLB expression in 1α-OHase^−/−^ mice supports the hypothesis that alterations in abundance or activity of molecules that regulate systolic and diastolic Ca^2+^ are centrally involved in depressed contractility in hypertrophied and failing hearts.

The key role of cardiac hypertrophy in the pathogenesis of heart disease underscores the need to identify the cellular and molecular mechanisms responsible for both cardiac hypertrophy and its progression to heart failure. A better understanding of disease progression may facilitate the development of novel therapeutic modalities, as well as the development of better guidelines for the prevention of cardiac hypertrophy. Our findings contribute to a better understanding of disease progression that are involved in vitamin D signaling and the development of heart failure. Our finding provides evidences that Ca^2+^ homeostasis and Ca^2+^ handling mediate structural and functional cardiac abnormalities in vitamin D deficient 1α-OHase^−/−^ mice and development of cardiac hypertrophy.

## Supporting Information

Table S1 **Comparison of cardiomyocytes resting sarcomere length in 0.5 Hz paced cells in WT and 1αOH^−/−^.** Sarcomere length of 1α-OHase^−/−^ and WT CMs, raw value in 0.5 Hz paced.
(DOCX)

Text S1 **Supplemental Information.**
(DOC)
