# Supplementary material for: Abnormal Calcium Handling and Exaggerated Cardiac Dysfunction in Mice with Defective Vitamin D Signaling
Source: PLoS One. 2014 Sep 30;9(9):e108382. doi: 10.1371/journal.pone.0108382 (PMC4182450; doi:10.1371/journal.pone.0108382)
Supplement: Table S1 — Comparison of cardiomyocytes resting sarcomere length in 0.5 Hz paced cells in WT and 1αOH−/−. Sarcomere length of 1α-OHase−/− and WT CMs, raw value in 0.5 Hz paced. (DOCX) [file pone.0108382.s001.docx]

**Table S1: Comparison of cardiomyocytes resting sarcomere length in 0.5Hz paced cells in WT and 1αOH^-/-.^**

| Resting Sarcomere length (lm) | | |
| --- | --- | --- |
|  |  |  |
| WT |  | 1αOH-/- |
| 1.7 |  | 2.25 |
| 1.67 |  | 2.27 |
| 1.47 |  | 2.21 |
| 1.57 |  | 2.3 |
| 1.81 |  | 2.33 |
| 1.59 |  | 2.24 |
| 1.73 |  | 2.31 |
| 1.75 |  | 2.26 |
| 1.68 |  | 2.24 |
| 1.58 |  | 2.32 |
| 1.68 |  | 2.41 |
| 1.78 |  | 2.3 |
| 1.8 |  | 2.31 |
| 1.71 |  | 2.27 |
| 1.72 |  | 2.25 |
| 1.66 |  | 2.29 |
| 1.64 |  | 2.28 |
| 1.57 |  | 2.25 |
