# Supplementary material for: Abnormal Calcium Handling and Exaggerated Cardiac Dysfunction in Mice with Defective Vitamin D Signaling
Source: PLoS One. 2014 Sep 30;9(9):e108382. doi: 10.1371/journal.pone.0108382 (PMC4182450; doi:10.1371/journal.pone.0108382)
Supplement: Text S1 — Supplemental Information. (DOC) [file pone.0108382.s002.doc]

##### SUPPLEMENTAL INFORMATION

##### Abnormal calcium handling and exaggerated cardiac dysfunction in mice with defective vitamin D signaling

Sangita Choudhury, PhD, Soochan Bae, PhD, Qingen Ke, MD, Ji Yoo Lee, BA, Sylvia S. Singh, MD, Federica del Monte, MD, Peter M. Kang, MD

**Supplemental Methods**

**Animal surgery**

TAC was performed in 8-10 week old male C57/Blk mice . After anesthesia with isoflurane (initially 4-5% in an induction chamber then 2% via intubation tube) via a precise vaporizer inhaler, the trachea was orally intubated and ventilated with a mouse ventilator (Model 687, Harvard Apparatus, Boston, MA) via a Y-shaped connector. The chest cavity was then opened, and the aortic arch was dissected with a 7-0 silk suture passed underneath the aorta. The ligation was done against a 25-G needle, which was then immediately removed to yield a patent, but stenotic aorta. The duration of the whole procedure was usually 15-20 minutes. The sham operation was a repeat of the procedure except for TAC.

#### Hemodynamic measurements

Baseline cardiac function was analyzed using echocardiography. Cardiac functional analysis was done 4 weeks after TAC by left ventricular (LV) pressure-volume loop measurement. Pressure-volume parameters were measured under isoflurane (2%) inhalant anesthesia using a 1.4-Fr microtip pressure-volume catheter (Scisense, Ontario, Canada), inserted into the right common carotid artery and advanced into the left ventricle. Data were recorded using a PowerLab system (ADInstruments, Colorado Springs, CO). Beat-by-beat pressure-volume parameters including stroke work, cardiac output, preload, afterload, and contractility were measured and analyzed using CardioSoft Pro software (CardioSoft, Houston, TX). Fractional shortening (FS) was measured as FS% = LVIDD – LVISD/LVIDD, where LVIDD is LV internal diastolic dimension and LVISD is LV internal systolic dimension. All measurements are averages of three cardiac cycles.

#### Morphometric analysis of isolated cardiomyocytes

The hearts from 12- to 14-wk-old male mice were retrogradely perfused with collagenase B and D (Sigma). The dissociated cardiomyocytes were plated on laminin (10 μg/ml)-coated dishes. After 1 hr of plating, unattached cells were removed by changing the media. Photographs were taken under a phase-contrast microscope, and cell surface area was determined by using the GNU Image Manipulation Program (GIMP 2.2.13) software (Open Source). Approximately 200 cells from each heart were measured, and the mean values for each heart were used for the statistical analysis.

**PTH level measurements**

The PTH level was measured in WT and in 1αOH^-/-^ before and after treatment with PC and vehicle. Using the mouse PTH 1-84 levels kit (immuneoptics Inc) the PTH level was measure from EDTA plasma according to the manufacturer instructions.

**CM isolation for Ca**^2+^ **transients and cell shortening experiments**

CMs were isolated from 2-month-old mice .The heart was excised, mounted on a Langendorff perfusion apparatus, and perfused with nominally Ca-free, *N*-2-hydroxyethylpiperazine- *N*8-2-ethanesulfonic acid (HEPES)-buffered Tyrode solution containing 1.5–2 mg/ml collagenase B and D (Sigma) until it started to become flaccid (10 min), after which tissue was separated, transferred to a flask containing fresh enzyme, and incubated further for 10–20 min. Free cells were separated, and the remaining undissociated tissue was reincubated (2 or 3 times if necessary) until most of the tissue had dissociated. The resultant cell suspension was rinsed several times, with [Ca^2+^]_o_ gradually increased to 1 mM. Myocytes were then plated onto laminin-pretreated coverslips.

**Field stimulation solutions and protocols**

CMs were continuously superfused with Tyrode solution at 37°C. The basic (normal) Tyrode solution (NT) contained (in mM) 140 NaCl, 10 glucose, 5 HEPES, 6 KCl, 1 MgCl_2_, and 1 CaCl_2_, adjusted to pH 7.4 with NaOH. Steady-state twitch Ca^2+^ transients were evoked by field stimulation at 0.5 Hz with platinum electrodes. Caffeine-induced contractures and Ca^2+^ transients were activated by rapid application of 10 mM caffeine in either NT solution or Na^+^- and Ca^2+^-free solution [0 Na^+^-0 Ca^2+^ solution; NT with Li^+^ replacing Na^+^ and 1 mM ethylene glycol-bis(baminoethyl ether)-*N*,*N*,*N*8,*N*8-tetraacetic acid (EGTA) replacing Ca^2+^]. The amplitude of these contractures and Ca^2+^ transients is an index of SR Ca^2+^ content. Because continuous application of caffeine prevents net SR Ca^2+^reuptake, the rate of [Ca^2+^]_i_ declines and relaxation during caffeine induced contracture, provides information about Ca^2+^ extrusion by Na^+^/ Ca^2+^ exchange (when in NT) or the combined action of the mitochondrial Ca^2+^ uniport and sarcolemmal Ca^2+^-ATPase (when in 0 Na^+^-0 Ca^2+^ solution). In measuring caffeine-induced contractures, we stopped steady-state stimulation for 5 seconds before caffeine solutions were introduced. For the caffeine-induced contracture in 0 Na^+^-0 Ca^2+^ solutions, the solution was first switched to 0 Na^+^-0 Ca^2+^for 10 seconds (to remove residual Ca^2+^) before caffeine application.

**Ca**^2+^ **transients and cell shortening measurements**

In field stimulation experiments, myocytes were incubated with 5 μmol/L Fura-2/AM (Molecular Probes), transferred to a Lucite chamber on the stage of an inverted microscope (Olympus1X70), and continuously superfused with Tyrode’s solution containing 1.2 mmol/L Ca^2+^. In all intact myocyte studies, shortening was measured using a video-edge detection system and stored using Ionwizard software (IonOptix LLC, Milton, MA). Sarcomere length (SL) and Ca^2+^ transient [Ca^2+^]_i_ were measured in myocytes stimulated at 5 Hz. The time-constant of relaxation for SL and [Ca^2+^]_i_ (τ) were used to estimate diastolic relaxation and Ca^2+^ removal. Caffeine (10 mmol/L) was rapidly infused after a 10-second pause following steady-state stimulation at increasing rates. All experiments were conducted at 37°C. SL was recorded with an IonOptix iCCD camera. Changes in average SL was determined by fast Fourier transform of the Z-line density trace to the frequency domain, and SL Shortening was calculated as follows: (diastolic SL-systolic SL)/diastolic SL. [Ca^2+^]_i_ was measured using the Ca^2+^ sensitive dye Fura-2 and a dual-excitation spectrofluorometer (IonOptix), alternately excited with a xenon lamp at wavelengths of 365 and 380 nm. The emission fluorescence was reflected through a barrier filter (510±15 nm) to a photomultiplier tube. The Fura-2 fluorescence ratio, the ratio of the photon live count detected by the excitation at 365 nm compared with 380 nm, represents [Ca^2+^]_i_. Autofluorescent backgrounds, measured on comparable sized cells from the same heart, were subtracted from each signal before the ratio F340/F380 was obtained. This ratio (R) was converted to [Ca^2+^]_i_, using minimum and maximum R values and the apparent dissociation constant (Kdb) which was determined in separate calibration runs. System background fluorescence was negligible.
